# Surgery's role in contemporary osteoarticular infection management

**DOI:** 10.3389/fped.2022.1043251

**Published:** 2022-12-19

**Authors:** Giacomo De Marco, Oscar Vazquez, Nathaly Gavira, Ardian Ramadani, Christina Steiger, Romain Dayer, Dimitri Ceroni

**Affiliations:** Pediatric Orthopedics Unit, Pediatric Surgery Service, University Hospitals of Geneva, Genève, Switzerland

**Keywords:** osteoarticular, infection, surgery, management, punction, drainage, necrotic, abscess

## Abstract

The treatment paradigm for osteoarticular infections (OAIs) has changed drastically over the past 80 years, from the advent of penicillin to the use of broad-spectrum antibiotics. Before these drugs, surgery was the only available treatment for OAIs; today, antibiotic therapy is considered the primary response to them. As a result, surgical treatment of OAIs is thus far more rarely indicated, sometimes even considered outdated and obsolete. However, long experience has taught us that many OAI contexts can still benefit from surgical management, constituting an essential complement to medical treatment. The present article seeks to contextualize this discussion by providing a chronological review of the surgical treatments used in cases of OAI and describing the quality of evidence supporting their rehabilitation in well-established situations.

## Introduction

Osteoarticular infections (OAIs) remain very serious affections; they can evolve into major morbidity, including disruptions to subsequent bone development, joint destruction, and permanent articular disability (1–3). Thus, ensuring that clinicians understand an OAI's etiology remains crucial to confirming the diagnosis, prescribing appropriate antibiotic therapy (4), and improving the outcome (5–7). Since the beginning of the 2000s, the widespread use of nucleic acid amplification assays (NAAAs) has substantially improved the detection of low levels of bacteriological agents in clinical samples and has significantly decreased the rate of “culture-negative” OAIs (8, 9). Better recognition of the microbiological causes of infections has drastically changed our understanding of the etiology of OAIs (5–7), concurrently with major changes in diagnostic approaches and therapeutic management.

Despite better overall knowledge of OAIs, some controversies remain concerning which clinical conditions should be treated using surgical procedures. Indeed, there is currently no obvious consensus about the treatment of OAIs, especially about which can be safely treated solely using medicines and which require and will benefit from a surgical approach. All pediatricians and pediatric orthopedists agree that an OAI's clinical presentation and probable outcome will differ depending on its microbiological causes; the treatments required may be diametrically opposed to each other. For example, OAIs due to pyogenic pathogens usually present with a more severe disease course, slower clinical response, and a potentially worse outcome. They may thus require invasive diagnostic and therapeutic procedures and rapid antibiotic treatment (10, 11). OAIs due to *Kingella kingae* (*K. kingae*), however, could theoretically be treated with antibiotic therapy alone (5). This literature review attempts to shed light on where to draw the line between cases of OAI for which surgical treatment seems to be the obvious solution and cases in which a surgical procedure would be useless or even excessive.

## Surgical procedures during OAIs: For whom, when, and how?

There are three priority questions when treating a child with an OAI. Does the child need surgery? Which surgical procedure should be considered? Must it be performed urgently? There are also three basic reasons for proposing a surgical intervention for pediatric OAIs: to obtain a bacteriological diagnosis, to ensure control of the infectious source, and to preserve the maximal function of the affected bone segment (12).

### Obtaining a bacteriological diagnosis

Identifying the pathogen responsible for an infection is essential since this is a precondition to tailoring a definitive antibiotic treatment regarding the therapy type, route, and duration (4). Since clinical outcomes may differ depending on the microbiological causes of an OAI, the first phase of a well-managed treatment is identifying the germ and understanding its characteristics. Indeed, identifying the incriminated pathogen will enable, in certain clinical situations, an exploration of whether it will express virulence factors such as antimicrobial genes, exotoxins, genes encoding microbial surface components recognizing adhesive matrix molecules, capsule genes, clonal complexes, or an accessory gene regulator (*agr*) group (13–18). Although a wide variety of pathogens can cause OAIs, the majority of complications and sequelae are due to *Streptococcus* species and *Staphylococcus aureus* (SA). Greater disease severity and complication rates have been described for bacterial isolates expressing the *agr III* and *agr IV* loci or exhibiting elevated vancomycin minimum inhibitory concentrations (13–18). In addition, studies from the mid-2000s suggested that MRSA was associated with a more severe infection phenotype than MSSA (19–22). Finally, in the past decade, the worldwide emergence of infections caused by Panton–Valentine leukocidin (PVL)-producing strains has been reported as responsible for multiple severe bone lesions (23). The risk of complications resulting from MSSA or MRSA PVL+ strains is high, with increased risks of subperiosteal abscesses, pyomyositis, and necrosing fasciitis, as well as a high incidence of deep vein thrombosis. Knowledge of pathogen characteristics is therefore crucial since the rate of orthopedic sequelae in the literature varies from 33% to 85%, depending on the strain responsible for the OAI (24).

Early recognition of the pathogen and its virulence factors may also condition the need and indication for complementary surgical treatment. Indeed, with certain pathogens (SA, *Streptococcus pneumoniae*), surgical treatment may be strongly recommended, if not even unavoidable, especially if they express specific virulence factors. On the contrary, OAIs due to less aggressive pathogens, such as *K. kingae*, should not require surgical procedures unless it is to exclude the involvement of pyogenic germs. Thus, the need to identify the pathogen responsible for an OAI seems to constitute a sufficient element for performing, at least, an arthrocentesis or a bone punction.

Numerous procedures have been developed to avoid unnecessary surgical procedures for bacterial diagnostic purposes. For children younger than 4 years old (in which more than 90% of OAIs are caused by *K. kingae*), an alternate, non-invasive diagnostic approach has been proposed involving taking an oropharyngeal swab and subjecting it to a sensitive molecular assay looking specifically for this microorganism. Compatible clinical and biological pictures, coupled with a positive *K. kingae*-specific NAAA from an oropharyngeal specimen, provide strong evidence that this microorganism is responsible for an OAI (25). However, it should be kept in mind that a positive test is not irrefutable proof of the disease's etiology since around 10% of young children carry the organism. Contrarily, failure to detect *K. kingae* deoxyribonucleic acid sequences with this sensitive molecular test virtually rules it out as the OAI's etiology (25). This diagnostic strategy is currently used efficiently and routinely worldwide with children under 4 years of age.

### Controlling the infectious source

Infectious source control is undoubtedly one of the main reasons for considering a surgical procedure. Bacteremia is quite common in acute pediatric osteoarticular infections and may be present in even more than 50% of the cases (26). Controlling the source of an infection necessarily involves preventing and treating bacteremia due to pyogenic pathogens, with a special mention for *S. aureus*. SA is currently considered as the leading cause of the bacteremia responsible for significant morbidity and mortality, and this regardless of the primary site of infection (27–31). SA bacteremia is, whatever its cause, frequently associated with severe metastatic infections, such as infective endocarditis, septic arthritis, osteomyelitis, and serious complications like sepsis and even septic shock—adverse outcomes that may prove challenging to manage (30, 31). Bacteremia The prevalence of metastatic infections among pediatric patients with SA bacteremia has been estimated to range between 29% and 52% (29). It has also been demonstrated that positive control blood cultures persisting beyond 48 h after the first blood culture triples the possibility of secondary sites of infection (28, 29, 32). Another study demonstrated that the onset of orthopedic complications was related to positive blood cultures and to the persistence of fever for more than 4 days, especially in children whose source control had been delayed (15).

As with most infections, bacterial sources of infection hold to the Latin aphorism “*Ubi pus, ibi evacua*”, meaning that wherever there is a collection of pus in the body, it should be evacuated as soon as possible, thus avoiding bacteremia, sepsis, and any subsequent complications. Thus, purulent synovial fluid, a bone or subperiosteal abscess, and necrotizing pandiaphysitis are cases in which surgical treatment is not an option but a therapeutic necessity. In these situations, isolated medical treatment will likely lead to therapeutic failure or complications. The best treatment will necessarily involve a surgical procedure aimed at substantially reducing the bacterial load by draining abscess collections and improving blood flow to the infectious site. This last parameter appears to be crucial since it will condition how well antibiotics penetrate infected and ischemic areas, leading to subpotent antibiotic concentrations and, thus, a reduced ability of antimicrobial agents to eradicate infection (33).

### Preserving maximal function of the affected bone segment

As functional impairment appears closely correlated to complications, the preservation of maximal function should focus on their prevention. The onset of complications and subsequent sequelae is thought to be related to inappropriate treatment of acute infections (34). Most authors consider diagnostic and therapeutic delays to be major risk factors for sequelae. Although a 4-day delay has been suggested as “acceptable” for the treatment of uncomplicated osteomyelitis, septic arthritis caused by pyogenic bacteria should be deemed an emergency and thus treated within the first 6–12 h of its onset (35).

Orthopedic complications after OAIs include chronic osteomyelitis, pathological fractures, partial or complete growth arrest with a limb length discrepancy and/or angular deformity, avascular necrosis, articular cartilage degeneration, chronic dislocation, and joint destruction. Few precise figures on the prevalence of post-infectious orthopedic sequelae are currently available. However, it is recognized that chronic osteomyelitis may develop in around 10% of acute osteomyelitis cases due to MSSA (36–39), whereas pathological fractures have been described in approximately 5% of cases sustaining hematogenous acute osteomyelitis (HAO) caused by the same pathogen (15, 40).

However, the worst complications are certainly those due either to an injury of the germinal cells (which can lead to irreversible growth arrest) or to avascular epiphyseal necrosis as a sequela of septic shock, articular septic joint tamponade, or infectious vascular insults. Penetration of the growth plate by a pathogen (transphyseal osteomyelitis) can affect the germinal cells of parts or the entire physis. This can lead to irreversible growth arrest (complete epiphysiodesis) or to an axial deviation of the limb (partial epiphysiodesis). As mentioned, avascular epiphyseal necrosis is probably the most serious issue in osteoarticular infections. Necrosis predominantly affects the epiphyses of large bones, such as the proximal ends of the femur and humerus. In sepsis and septic shock, the normal anticoagulative state within the epiphyseal vasculature is disrupted, resulting in a hypercoagulable state characterized by microvascular thrombi, fibrin deposition, neutrophil extracellular trap (NET) formation, and endothelial injury. Thus, blood shunt and prolonged hypo-oxygenation during every phase of shock and multiorgan failure may explain the hypoperfusion of the epiphysis responsible for avascular necrosis, growth arrest, and epiphyseal dysmorphism. Although these complications were initially linked to meningococcemia, they are now associated with septic shock events caused by other organisms (e.g., Pneumococcus, gram-negative bacilli, Streptococci) that occur in pediatric populations, especially when the infectious source is initially badly controlled.

#### Surgical treatment of septic arthritis for maximal joint preservation

The course of septic arthritis, whether treated or not, varies substantially depending on the germ that causes it. In cases of septic arthritis caused by a pyogenic microorganism, the clinical course is characterized by an inflammatory cascade. This results in a huge influx of immune cells and neutrophils to the infection site, macrophage activation, and the release of inflammatory cytokines such as IL-1B, IL-16, TNF-a, MIP-2, and granulocyte-macrophage colony-stimulating factor (GM-CSF) (41, 42). Phagocytosis of bacteria by macrophages, synoviocytes, and polymorphonuclear leukocytes will release more lysosomal enzymes and reactive oxygen species and will further induce the liberation of cytokines (43–45). The high levels of cytokines finally induce the release of host matrix metalloproteinases and lysosomal enzymes, which are responsible for further joint degradation (46, 47). It is recognized that cartilage destruction starts to occur as early as 8 h after infection (48), even if intravenous antibiotic therapy is started within the first 24 h of infection, since significant glycosaminoglycan destruction and collagen disruption may be noted very early on during septic arthritis.

Even more worryingly, the antigen-induced inflammatory response may persist and continue to damage the joint architecture after the infection has been cleared (49, 50). In addition to enzymes from bacteria, white cells, and synovium, the cartilage's own chondrocytes may also play a part in its destruction (51). An ultrastructural analysis of chondrocytes in experimentally produced septic arthritis showed an increase in lysosomal electron-dense bodies, suggestive of the production of proteolytic enzymes in both the superficial and deep layers of articular cartilage (51). In addition, it is now recognized that chondrocytes respond to IL-1 by breaking down the surrounding proteoglycan matrix, even in mature cartilage without sepsis (52–54).

However, septic arthritis due to less aggressive pathogens, such as *Kingella* or *Moraxella* species, only presents with mild clinical signs and few moderate laboratory findings. When bacteriological diagnostic is certain, most septic arthritis due to these germs would benefit from antibiotic treatment alone without additive surgical procedures.

Recognition of the causal germ is essential because it will condition subsequent therapeutic management. Thus, given the pathophysiological mechanisms of cartilage destruction, it would seem irrational not to carry out surgical irrigation and debridement of an infected native joint, especially when pyogenic pathogens are suspected or clearly involved. The mode of surgical treatment for septic arthritis is itself a source of controversy involving which joint is affected, the suspected pathogen, and the patient's age. Drainage and lavage of a septic joint may be performed rapidly either using a simple syringe injection–aspiration procedure for septic arthritis with minimally inflammatory joint fluid (55), or using arthrotomy, or arthroscopy for joints with significant purulent effusion (56–59).

#### Surgical treatment of hematogenous acute osteomyelitis (HAO) for preserving bone structure and function

It is important to remember that before the development of antibiotics, surgery was considered the only therapy available for treating HAO. Surgical procedures revolved around coherent concepts such as trepanation for facilitating pus drainage (60, 61), decreasing the intraosseous pressure responsible for bone infarction, and, above all, debridement to remove all infective and necrotic material (62, 63). Following the introduction of penicillin and broad-spectrum antibiotics, reports described drastically reduced mortality rates; however, many authors noted that the incidence of complications (especially the development of chronic osteomyelitis) was very high when using penicillin alone with the usual supportive measures but without surgical drainage (64, 65). Indeed, these authors concluded that, despite the use of effective antibiotic treatments, surgical treatments still had a crucial role to play, and their experiences resulted in an acceptance that many specific criteria remained formal indications for surgical procedures. They described clinical situations, still relevant today, for which surgical intervention seemed to be appropriate (64, 65). Their guidelines recommended that soft tissue abscesses had to be incised and drained, as did subperiosteal abscesses. Interestingly, they believed the subperiosteal space should be considered as a compartment and that the pressure exerted in that space was considerable in cases of HAO, which could lead to an equivalent of compartment syndrome, interrupting circulation and producing extensive necrosis of the bone (64, 65). Their thinking revolved around the crucial concept that antibiotics could not prevent permanent bone damage once blood supply had been interrupted (64, 65). The guidelines also suggested that metaphyseal bone abscesses should be incised and drained and that metaphyseal bone decompression could decrease the bone pressure capable of interrupting circulation and producing extensive necrosis (64, 65). Finally, they recommended that the medullary canal should be decompressed in patients with continuing bone pain, sepsis, or failure to respond to antibiotic therapy.

Surgical management of HAO remains challenging. Despite abundant publications on this subject, debate continues between those recommending surgery and those considering it excessive. Unfortunately, the debate focuses on whether or not to perform a therapeutic surgical procedure, whereas the right question would be which cases of HAO and, above all, which pathogens require it.

There is growing evidence that surgical drainage is not an option, but it may appear as a real obligation, especially when radiological investigations highlight the presence of pus in the soft tissues, the subperiosteal space, the diaphysis cavity, or even the metaphysis (64–69). Surgical management's role in cases of HAO should be to improve the local environment for antibiotic delivery by decompressing and washing the subperiosteal/metaphyseal abscess cavity, decreasing the bacterial load, and removing devitalized necrotic tissue, thereby preventing the progression of infection to chronic osteomyelitis and lesions of the physis/epiphysis (64–69). Adequate debridement of devitalized tissue can lead to a large bony defect, also called dead space, whose management is crucial, with the objective being to replace dead infected bone with vascularized tissue. The specifications for surgery, therefore, include 6 objectives that medical treatment cannot provide (64–69):

- Draining/decompressing the bone and subperiosteal space
- Reducing the bacterial load
- Removing necrotic tissue
- Improving bone vascularization
- Avoiding physeal/epiphyseal lesions
- Preventing evolution toward chronic osteomyelitis

Surgery thus remains a cornerstone of treatment for children with HAO with subperiosteal, soft tissue, or bone abscesses, especially when they present late after the onset of symptoms. In these cases, prompt surgical decompression with sequential intravenous antibiotics gives the maximum chance of an excellent outcome.

Finally, it is important to note that even the greatest study which validated the medical treatment of osteomyelitis reported the need for surgical procedures; in fact, Peltola et al. affirmed in their paper that “*surgery was kept at minimum*” and that “*most children underwent only the diagnostic percutaneous aspiration or drilling, and only 24% had no surgery*” (70).

## Radiological investigation: The key to tailored surgical treatment?

Indications for the surgical treatment of OAIs also suggest an urgent need for high-quality imaging, which is of paramount importance to confirm the diagnosis, define the degree of infection, plan surgical procedures, and control the response to therapy. Magnetic resonance imaging (MRI) is currently considered the most highly sensitive means of investigating and detecting OAI, especially in its early days. MRI is also probably the best means of detecting soft tissue abscesses. Gadolinium-enhanced sequences help to outline zones of necrosis (71), and are useful for detecting abscesses, whether subperiosteal or intraosseous (67). However, recent reports, demonstrating pathological retention in brain tissue of children exposed to gadolinium-based contrast agents (GBCAs) should encourage radiologists to avoid its unnecessary use in pediatric patients (72–74). MRI can also help to plan treatment, particularly the percutaneous drainage of fluid collections and surgical debridement; it enables an assessment of the extent of necrotic tissue and the definition of dangerous contiguous structures that will need customized management to avoid morbidity and complications (75). In addition, contrast-enhanced sequences of MRI can be used to clearly identify extension of the infection into soft tissues or to detect real childhood pyomyositis.

Thus, we believe it is reasonable to assume that, alongside recognizing the infection's causative germ, MRI is a decisive element in deciding on the need for a surgical procedure.

Emerging technologies such as positron emission tomography (PET)–MRI should improve the diagnosis of bone infections even more (76). However, hybrid PET–MRI scanners are very expensive, and their widespread clinical implementation thus remains problematic. It seems preferable to use MRI as a primary tool for uncomplicated unifocal cases, whereas cases with (potentially) multifocal disease, or a contraindication for MRI, could prefer PET.

## Conclusion

The early diagnosis of OAI and the commencement of antimicrobial treatment are crucial to good patient outcomes, as is the control of the infectious focus. This therapeutic approach may be sufficient in certain cases, but it took being risky to want to generalize them to all OAI. In fact, many OAI still require surgical procedures for their diagnosis, focus control, and function preservation.
