# Erratum: Surgery's role in contemporary osteoarticular infection management

**DOI:** 10.3389/fped.2023.1170859

**Published:** 2023-03-02

**Authors:** Frontiers Production Office

**Affiliations:** Frontiers Media SA, Lausanne, Switzerland

**Keywords:** osteoarticular, infection, surgery, management, punction, drainage, necrotic, abscess

An Erratum on Surgery’s role in contemporary osteoarticular infection management By De Marco G, Vazquez O, Gavira N, Ramadani A, Steiger C, Dayer R and Ceroni D. (2022) Front. Pediatr. 10:1043251. doi: 10.3389/fped.2022.1043251

An omission to the funding section of the original article was made in error. The following sentence has been added: “Open access funding was provided by the University Of Geneva”.

The original version of this article has been updated.

