# Supplementary material for: Surgery's role in contemporary osteoarticular infection management
Source: Front Pediatr. 2022 Dec 19;10:1043251. doi: 10.3389/fped.2022.1043251 (PMC9806351; doi:10.3389/fped.2022.1043251)
Supplement: Supplementary file 1 [file Table1.docx]

Conditions suggesting realization of a surgical procedure

1. Obtaining a bacteriological diagnosis
2. Controlling the infectious source
   - Draining a bone’s abscess
   - Draining a subperiosteal abscess
   - Draining a soft tissue abscess
   - Removing necrotic tissue
3. Avoiding antigen-induced inflammatory response into the joint
4. Improving bone vascularization
5. Improving bone vascularization
6. Avoiding physeal/epiphyseal lesions
7. Preventing evolution toward chronic osteomyelitis
